# How is the effectiveness of immune surveillance impacted by the spatial distribution of spreading infections?

**DOI:** 10.1098/rstb.2014.0289

**Published:** 2015-08-19

**Authors:** Ulrich D. Kadolsky, Andrew J. Yates

**Affiliations:** 1 Department of Systems and Computational Biology, Albert Einstein College of Medicine, 1300 Morris Park Avenue, Bronx, NY 10461, USA; 2 Department of Microbiology and Immunology, Albert Einstein College of Medicine, 1300 Morris Park Avenue, Bronx, NY 10461, USA; 3 Institute of Infection, Immunity and Inflammation, Glasgow Biomedical Research Centre, University of Glasgow, 120 University Place, Glasgow G12 8TA, UK

**Keywords:** computational immunology, cytotoxic T cells, spatial modelling

## Abstract

What effect does the spatial distribution of infected cells have on the efficiency of their removal by immune cells, such as cytotoxic T lymphocytes (CTL)? If infected cells spread in clusters, CTL may initially be slow to locate them but subsequently kill more rapidly than in diffuse infections. We address this question using stochastic, spatially explicit models of CTL interacting with different patterns of infection. Rather than the effector : target ratio, we show that the relevant quantity is the ratio of a CTL's expected time to locate its next target (search time) to the average time it spends conjugated with a target that it is killing (handling time). For inefficient (slow) CTL, when the search time is always limiting, the critical density of CTL (that required to control 50% of infections, C^*^) is independent of the spatial distribution and derives from simple mass-action kinetics. For more efficient CTL such that handling time becomes limiting, mass-action underestimates C^*^, and the more clustered an infection the greater is C^*^. If CTL migrate chemotactically towards targets the converse holds—C^*^ falls, and clustered infections are controlled most efficiently. Real infections are likely to spread patchily; this combined with even weak chemotaxis means that sterilizing immunity may be achieved with substantially lower numbers of CTL than standard models predict.

## 1. Introduction

CD8^+^ cytotoxic T lymphocytes (CTL) are key elements of the adaptive immune system in vertebrates and are critical in resolving or limiting many infections, including human and simian immunodeficiency viruses (HIV/SIV) [1–4], hepatitis C [5,6], lymphocytic choriomeningitis virus (LCMV) [7,8], influenza [9–11] and listeria [12,13]. After encountering and recognizing an infected cell by binding pathogen-derived peptides presented on MHC class I molecules on the cell surface, a CTL may lyse the cell directly, or indirectly through the recruitment of phagocytic cells [14]. CTL may also suppress an infection through non-lytic, cytokine-mediated mechanisms [15,16].

It has recently become clear that populations of T cells (tissue-resident memory cells, or T_rm_) continually survey potential sites of pathogen entry or reactivation and are able to re-acquire effector function rapidly [17–19]. Cytotoxic T_rm_ are likely to be crucial for sterilizing immunity to pathogens such as HIV that enter through mucosal surfaces and become very difficult to control once disseminated throughout the body. However, the local densities of CTL needed to control a given infection are unknown. A model-guided estimate of this quantity would be useful for establishing the feasibility of vaccines aimed at inducing tissue-resident CTL memory—if only to assess whether the required densities are physiologically possible.

For purely CTL-mediated control, the net growth rate of an infection is the balance between its rate of spread among susceptible cells and the total rate at which CTL can kill infected cells. We will use C* to denote the minimum (or critical) density of CTL required to reduce the net growth rate of an infection to zero. One approach to estimating C* is to use *in vitro* experiments to estimate the minimum effector : target (E : T) ratio needed for suppression of infected cell growth [20]. Another means is to begin with measured microscopic quantities such as tissue-specific rates of CTL movement or surveillance rates—the number of potential targets one CTL can survey per unit time. These parameters can be inferred from intravital microscopy or *in vivo* CTL killing assays, described below, and used as inputs to dynamical models of CTL interacting with infected cell populations. One such model, used extensively to describe the within-host dynamics of viral infections, assumes ‘mass-action’ kinetics in which the rate of loss of infected cells is linear in both CTL and infected cell numbers. That is, for a population of infected cells growing at net rate *r* in the absence of CTL, and with a CTL population *C* killing these cells,1.1
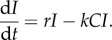


When *C* is expressed as a proportion of all surveyable cells, the interpretation of the rate constant *k* is the mean number of cells of all types (infected or not) that one CTL surveys per unit time, multiplied by the probability of recognition and killing of an infected cell following contact with it [21]. Mass-action has been shown to describe CTL killing of melanoma cells both *in vitro* and *in vivo* [20]. It also gives reasonable descriptions of assays in which peptide-pulsed cells are injected intravenously, migrate to the spleen and are killed by peptide-specific resident CTL. In the studies that enumerated cell populations in spleens directly, using LCMV [22–25], polyoma virus [26] and influenza virus [21], *k* was estimated to be in the range 0.14–4 cells min^−1^. Graw *et al.* [27] performed longitudinal sampling of target cells in blood to infer the dynamics of CTL and targets in the spleen, and estimated *k* to be between 8 and 35 cells per minute. The reasons underlying this two-orders-of-magnitude spread are unclear. Differences in *k* may derive from differences in CTL motility, in the spatial distribution of CTL and targets within the spleen and so the numbers of CTL effectively participating in clearance of targets, and in the efficiencies of killing following conjugation [21].

Three assumptions underlying equation (1.1) are that (i) CTL and the cells they survey are well mixed and that encounters of one CTL with infected cells occur with Poisson statistics; (ii) CTL perform undirected random walks (i.e. without preferential motion towards infected cells, referred to as chemotaxis) and (iii) the time a CTL spends conjugated with an infected cell, the handling time, can be neglected. The first assumption holds when infected cells and CTL are scattered diffusely throughout a tissue. The second is likely to be satisfied in the splenic CTL killing assays, because levels of inflammation will be low with peptide-pulsed (uninfected) targets and so substantial chemotactic bias in CTL movement seems unlikely. The third assumption is expected to be valid when the handling time *h* is very short compared with 1/(*kI*), which is the expected time taken for a CTL to locate its next infected cell, when these are present at spatial frequency *I*, and/or when E : T is very large so very few CTL are expected to kill more than once in order to bring an infection under control.

If these assumptions hold, then with knowledge of the growth rate *r* and surveillance rate *k*, the critical CTL density is then simply C*= *r*/*k*. The simple mass-action model can be extended to explicitly include, for example, the time taken for CTL to kill infected cells (the handling time), the dynamics of virus epitope expression on infected cells and the eclipse phase between infection of a cell and virus production [28–30]. Closed-form solutions for C* may not be available in these models, but C* can be obtained numerically [28].

However, it is not obvious that equation (1.1) or its extensions are appropriate descriptions of CTL interacting with live replicating intercellular pathogens, for at least two reasons. First, a mass-action model assumes that infected cells will appear at random within an infected tissue, but pathogen transmission from cell to cell likely occurs preferentially over short ranges and as a consequence infections of relatively immobile cells will tend to spread in foci (e.g. [31–33]). Second, CTL may be attracted to areas of infection and cell death via chemokines, breaking the assumption that CTL perform unbiased random walks. So using mass-action models to describe CTL killing in such situations may lead to incorrect estimates of critical CTL densities. Assuming we know the microscopic quantity *k*, the uncontrolled pathogen growth rate *r*, and the handling time *h*, what impact does the spatial distribution of targets, and the degree of chemotaxis, have on the critical density of CTL needed for immunity? Here we use explicitly spatial stochastic simulations of infections to address this question.

## 2. Methods

We developed a stochastic agent-based model (ABM) representing the behaviour of CTL surveying a tissue containing a spreading population of infected cells. The ABM was explicitly spatial and allowed us to vary the patterns of infection spread, track CTL-target conjugate dynamics and implement chemotaxis of CTL towards infected cells. The simulator was written in C/C++ and derived in part from the open source software Scriptbots. It is freely available at https://github.com/udkad/immunebots.

### (a) Overview of the agent-based model

The simulation is set up as a two-dimensional grid of non-susceptible, susceptible and infected cells. In all simulations, cells were uniformly placed in a two-dimensional matrix of 320 rows and 320 columns (total cells: 102 400). All cells were susceptible to viral infection. The grid size corresponds to a physical area of 3.2 mm by 3.2 mm (≈10 mm^2^) with toroidal (wrap-around) boundary conditions. This choice of boundary conditions mirrors a situation in which the simulated infection site is surrounded by others with similar statistical properties. All cell types and virions produced by infected cells are modelled individually as agents with internal variables corresponding to age, time since infection, time since last lytic event, etc*.* Susceptible cells are immobile and are infected by virions upon contact. Infected cells are also immobile, produce virions and die due to viral cytotoxicity (half-life of approx. 1.4 days) or due to CTL lysis. CTL exhibit three different kinds of behaviour: (i) searching for cells with a random walk (either undirected, or with biased movement in response to chemotactic cues), (ii) scanning cells upon contact or (iii) remaining in conjugates with infected cells after scanning and while lysing them. Virions travel in a straight line from their parent cell in a randomly chosen direction until they are cleared (half-life of approx. 4 h) or infect a susceptible cell. This simplified virion movement was chosen simply as a means to generate infections spreading in clusters and not to accurately represent mechanisms of intercellular transmission. Upon contact with a susceptible cell, the virion immediately infects the cell. At 1 s intervals all agents are updated with information regarding their current environment, and choose an action based on the available actions in the current state with probabilities weighted by the associated rate constants. CTL and virions move unobstructed on the two-dimensional plane with positions recorded at floating-point precision, but coarse-grained to a scale of 1 μm for the detection of contacts. Multiple CTL can bind unhindered to a single living target, but lysis time remains unaffected by multiple bindings. A single CTL is assumed to be able to kill multiple times. Rate constants and other parameters used for the simulation are detailed in the electronic supplementary material, table S1.

### (b) Controlling the infected cell growth rate

In order to compare the ability of CTL to control different patterns of infection, it was necessary to fix the uncontrolled growth rate of infected cells. We adjusted the parameters for diffuse and clustered infections to ensure that infected cell numbers grew exponentially with rate constant *r* = 1 d^–1^ in the absence of CTL, corresponding to a doubling time of ln(2)/r ≃ 17 h. This is roughly comparable to the very early rate of growth of SIV infection in the gut lamina propria in rhesus macaques [28,31]. Setting the growth rate was straightforward for the case of diffuse infections, designed to mimic the spatial distribution of target cells implicit in mass-action infection dynamics; in each short time interval *δt* ≪ 1/*r* every infected cell had a probability *r*δ*t* of infecting a susceptible cell chosen at random, regardless of its physical distance. Modelling the spatial dynamics of virions was not required in this scenario. For clustered infections, the growth rate is determined by a combination of the virion production rate, speed and rate of clearance. To achieve a net growth rate *r* = 1 d^−1^, these parameters were sampled from a uniform distribution from ranges obtained from the literature [34–38]. The parameter which most strongly influenced the rate of growth was the virion production rate. This parameter was then varied by ±5% in steps of 0.005%, and each parameter set was simulated 1000 times. The infected cell growth rate was calculated from each simulation, and then linear regression was used to find the virion production rate which corresponded to an overall growth rate *r* = 1. Parameters used for diffuse and clustered infections are shown in the electronic supplementary material, table S1.

### (c) CTL speed and conjugate dynamics

Two parameters in the ABM govern the CTL surveillance rate (the rate at which CTL move between surveyable cells, in the absence of killing): CTL velocity, the straight-line distance moved in micrometres per second; and the scan time, the time a CTL spends stationary whilst scanning an uninfected cell. To achieve a prescribed surveillance rate in the simulation, 100 CTL were placed among uninfected, non-susceptible cells. Multiple simulations were run with different parameter sets. For CTL velocity, the simulated range was 1–35 μm min^−1^ [39,40]. Mempel *et al*. [41] estimated that detachment without lysis following an encounter with an infected cell takes approximately 2 min. This is therefore a strong upper bound on the scanning time. Because the majority of the *in vivo* peptide-pulsed target killing assays give estimates of the order a minute for the mean time spent between contacts, we used CTL scanning times of between 1 and 15 s cell^−1^. Achieving such a rate of surveillance of the order 1 min required relatively fast-moving CTL with short scan times. We used a velocity of 7.5 μm min^−1^ and a scan time of 5 s, which translated to a CTL surveillance rate of 1.1 cells min^−1^. To study the control of infections at high E : T or search to handling time (S:H) ratios, CTL speed was reduced to 0.18 μm min^−1^, which translated to a CTL surveillance rate of 0.022 cells min^−1^. Finally, CTL may remain attached to infected cells for tens of minutes [41,42]; in our simulations we used a handling time of 30 min.

### (d) Modelling CTL chemotaxis

Chemotaxis was implemented as follows. Every time the CTL moves in a new direction, the new direction angle is chosen either at random from the directions ±45° or the direction of the nearest infected cell. This choice occurs when a CTL comes to the end of a persistent segment of its motion (default length of 25 μm), finishes scanning an uninfected cell or lyses an infected cell. Note that although CTL motion has a persistence length, the high density of cells means that a CTL ‘step’ is almost always interrupted by a scan event. The strength of chemotaxis was determined by the frequency of non-random movements. Frequencies used in the simulations were 0% (no chemotaxis), 1%, 5% and 20%. CTL under directed motion are attracted to the nearest living target, which included CTL-target conjugates. Varying levels of chemotaxis did not influence the cell speed, such that the surveillance rate *k* remained constant.

### (e) Estimating the critical CTL density C*

In the deterministic case, C* is the CTL density required to reduce the growth rate of the infection to zero. In the stochastic case, we define C* as the density of CTL (as a fraction of all scannable cells) required to drive the virus extinct in 50% of simulations. For a given *C*, our estimate of the probability of extinction was the proportion of 200 simulations in which the infected cell count fell to zero. Where a clear determination was not possible, for example when the infected cell frequency fluctuated near zero, the outcome was determined by the gradient of the time course of infected cell numbers over the last 12 h of the simulation—negative implies eventual extinction, positive implies a successful infection. We used a simple adaptive search algorithm to identify C* given broadly spaced initial guesses. The dependence of the probability of extinction on *C* was well described with a sigmoid function2.1
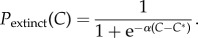
The above equation was then used to estimate the free parameters C* and the steepness parameter *α*, using the Nelder–Mead algorithm in the FME package in *R* [43].

## 3. Results

### (a) The agent-based model recapitulates deterministic mass-action models when targets are spread diffusely

We wanted to assess the effect of spatial structure of infected cell populations on the ability of CTL to control infections. The critical CTL density C* predicted by the simplest canonical ordinary differential equation (ODE) model might be used as a first guess of the CTL required and serves as a reference point. With this in mind, we performed a calibration process to ensure that the average dynamics predicted by the stochastic ABM simulations of diffuse infections (see Methods), in which CTL and infected cells are well mixed, agreed with those predicted by simple mass-action models.

We compared the simulations to two deterministic models. The first is the simplest mass-action model (equation (1.1)) describing CTL killing of target cells *T* replicating at uncontrolled rate *r*3.1
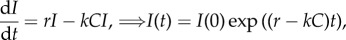
where *I* and *C* are expressed as dimensionless quantities in the range [0,1]. Recall that we assume 100% efficiency of killing of targets following contact *k* is the surveillance rate, such that 1/*k* is the mean time for a CTL to move between one cell of any kind and the next; and 1/(*kI*) is the search time, or the expected time for a CTL to locate an infected cell. The critical CTL density is then C* = *r*/*k*; in this model, densities greater than this guarantee eventual clearance of the infection.

The second we call the extended mass-action model. Upon recognition of an infected cell, CTL may remain attached for tens of minutes [41,42]. An appropriate deterministic model that includes this process is an age-structured partial differential equation that follows the population density of CTL and infected cells that have been conjugated for a time *τ*, *X*(*t*,*τ*)3.2
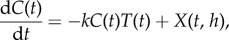
3.3
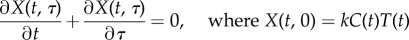
3.4
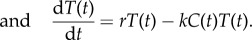
The extended mass-action model reduces to the simpler model when CTL are in excess (high E : T, or equivalently *C*_0_ ≫ *T*(0)) and/or when handling times are short compared with search times, *h* ≪ 1/(*kT*), such that the age structure in equation (3.3) can be neglected [28].

To perform the comparisons, we prescribed the handling time (*h* = 30 min), the infected cell growth rate in the absence of CTL (*r* = 1 d^–1^), the time taken to scan an uninfected cell (which was assumed to be negligible and not considered in either deterministic model) and the surveillance rate (*k*) as inputs to the ABM. The infected cell population was allowed to grow to a size of 1000 cells distributed across a grid of 10^5^ cells in total before CTL were introduced spatially at random at known densities *C*. We simulated conjugates persisting for a fixed handling time *h* = 30 min.

**Figure 1.**
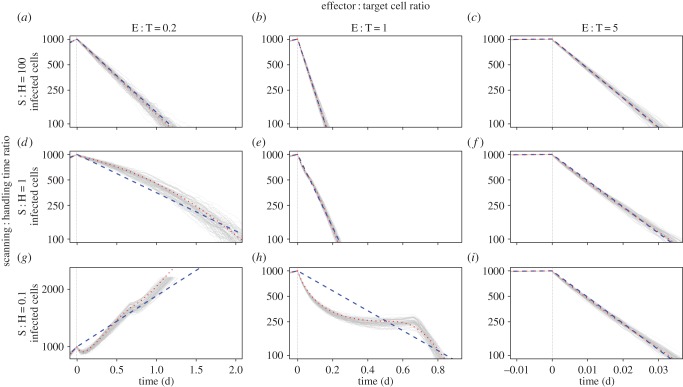
Kinetics of infected cells spreading diffusely and being killed by CTL. Infected cells (vertical axes, logarithmic scale) were seeded in the simulation and allowed to grow to 1000 cells, appearing at random within the tissue, before CTL were introduced randomly among them, at a time denoted day 0. We varied the ratio of CTL to effector cells at day 0 (horizontally) and the ratio of search time to handling time (vertically) to explore the ability of the simple mass-action (dashed blue line) and extended mass-action (dotted red line) ODE models to explain the simulated data. Note how the extended model better fits the data when the handling time is greater than the search time (lower left and lower centre figures).

We found close agreement between the mean timecourse of diffuse infection simulations and the extended mass-action model, for all parameter regions (figure 1). As expected, the simple mass-action model held at high S : H and/or high E : T but broke down when both conditions were violated. However, given noise in experimental data, apparently mass-action dynamics (linear decay of infected cells on log scale) might superficially appear to hold at low E : T (figure 1, left hand column), as has been observed experimentally [25].

### (b) The influence of the spatial distribution of targets on efficiency of CTL control

Next we wanted to assess the impact of breaking the assumptions of the simple mass-action model on critical CTL densities. We began by preserving the assumption that CTL perform undirected random walks, and compared their ability to control two modes of pathogen spread: one in which infected cells appear randomly and are uniformly distributed across the tissue, and a clustered model in which infection spreads in foci (see Methods). In both cases, the tissue was seeded with a single infected cell and the infection was allowed to grow to different cell numbers *I*_C_ before introducing CTL distributed at random across the tissue. For each value of *I*_C_, we performed repeated simulations with different numbers of CTL (*C*) to estimate the probability of extinction as a function of *C*. As described in Methods, the critical density C* was the value of *C* at which 50% of infections eventually went extinct.

We used two values of the surveillance rate, *k*, in order to explore a wide range of S : H and E : T ratios without computational expense becoming prohibitive at large numbers of infected cells. Control at high S : H was explored using *k* ≃ 0.02, substantially lower than the values estimated in *in vivo* killing assays. However, the parameter *k* is implicitly the product of the surveillance rate and the probability of recognizing a cell as being infected and initiating lysis, so this value of *k* may correspond to inefficient (or perhaps exhausted) CTL. Control at low E : T was explored with *k* ≃ 0.7, a value derived from *in vivo* measurements of CTL velocities and which is in line with the estimates from splenic killing assays. Critical CTL densities were expressed in units of the basic mass-action estimate C* = *r*/*k*, which allowed us to compare the fold-changes in critical CTL densities for different spatial distributions of targets across different values of *k*.

#### (i) When search times are much longer than handling times, the spatial distribution of targets has little impact on the efficiency of CTL, and mass-action provides good estimates of C*

**Figure 2.**
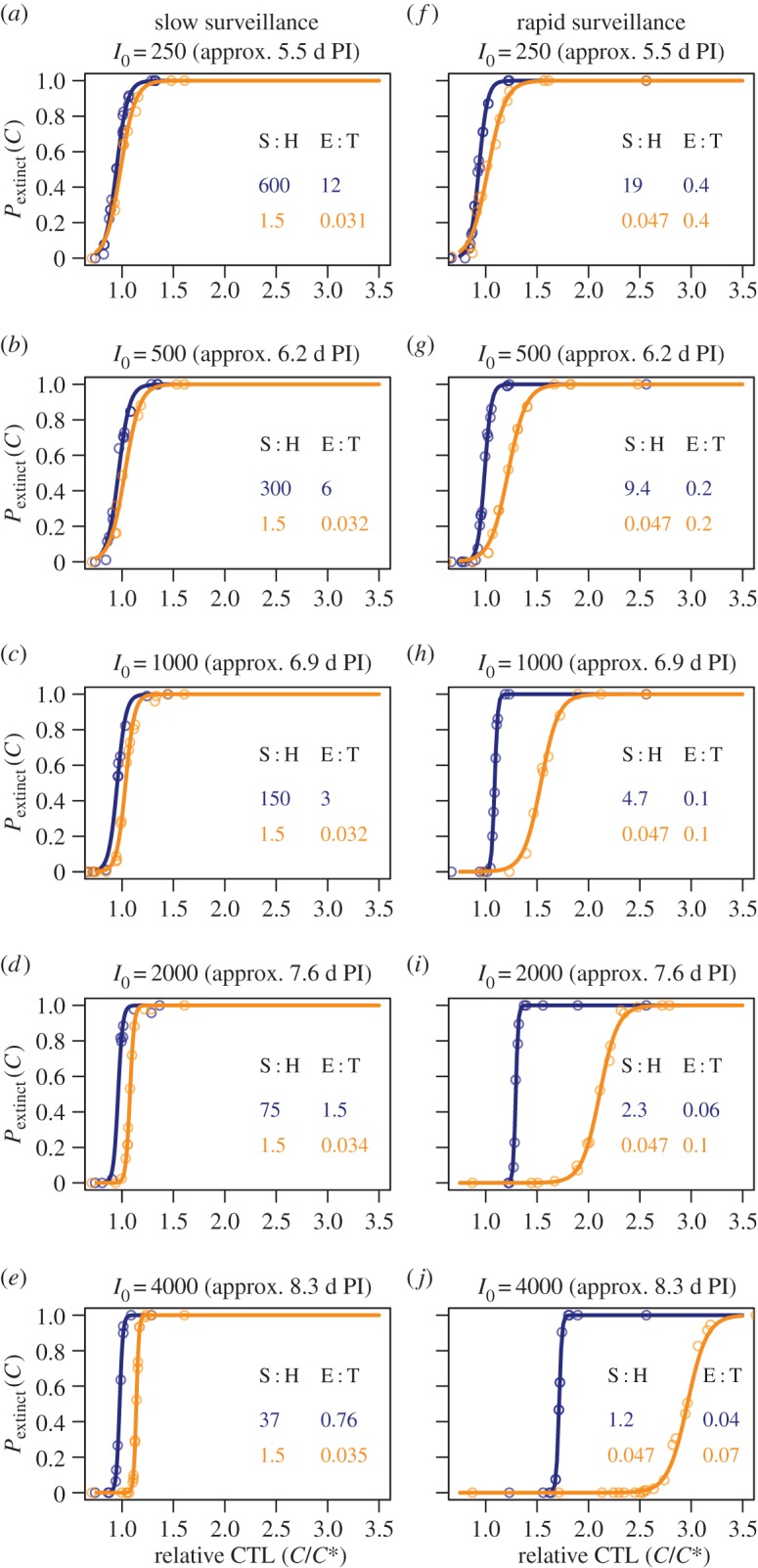
Probabilities of extinction as a function of CTL density in units of the mass-action estimate C*= *r*/*k*. (*a–e*) Slow CTL surveillance such that S : H > 1 even within packed clusters of infected cells; (*f–j*) more rapid CTL migration, such that S : H < 1 within clusters. Blue curves, diffuse infections; orange curves, clustered infections. S : H ratios are approximate and calculated as follows: for diffuse infections, *S* is the expected time for a CTL to locate its first target following the appearance of CTL when infected cell numbers have reached *I*_0_, *S* ≃ *N*/*kI*_0_, where *I*_0_/*N* is the proportion of all susceptible cells infected. For clustered infections, the search time is quoted as the time taken to move between adjacent cells within a packed cluster of infected cells, and so is a strong lower bound on the average search time. E : T ratios are quoted as the ratio of total effectors to total targets at the critical CTL density C*. PI, post-infection.

At the low surveillance rate *k* ≃ 0.02, C* for diffuse infections was close to the mass-action prediction (C* ≃ *r*/*k*, or relative C* ≃ 1) for initial infected population sizes of between 250 and 4000, corresponding to CTL appearing between 5.5 and 8.3 days post infection (figure 2*a–e*, blue curves). The stochasticity in infection dynamics in this model is reflected in the sigmoid shape of these curves—extinction is possible even for CTL densities below the critical level predicted by the deterministic model.

Strikingly, at this low surveillance rate we found that breaking the well-mixed assumption and allowing infections to be clustered had very little impact on extinction probabilities (figure 2*a–e*, orange curves) with departures only becoming apparent when E : T fell below 1 (figure 2*e*).

This insensitivity to spatial structure derives from the ratio of search to handling times. Recall that mass-action is expected to hold when the E : T ratio is high and/or the mean time for a CTL to locate its next target, 1/*kI*, is greater than the handling time *h*. For diffuse infections, even up to day 8 when 4000 cells are infected out of 10^5^ potential targets (figure 2*e*), the search time 1/*kI* ≃ 830 min is much longer than the handling time, and so we predict and observe simple mass-action kinetics with C*= *r*/*k*, even if E : T falls below 1 (figure 2*a–e*, blue curves). Second, if this low level of *k* reflects low motility rather than low detection efficiency, control of clustered infections is likely mediated predominantly by CTL resident in or near foci of infection. Even within a large densely packed cluster in which 100% of cells are infected, at this low *k* the mean search time is still 1/*k* ≃ 50 min, compared to a handling time of 30 min. If CTL and targets within a cluster are well mixed, then if handling time is comparable to search time, simple mass-action is expected to hold asymptotically [28], giving C* ≃ *r*/*k* (figure 2*a–e*, orange curves).

We conclude that when CTL perform undirected random walks and are relatively inefficient such that the mean time to move between infected cells is greater than or of the order of the handling time, even within foci of infection, the spatial distribution of targets has little impact on C* and it lies close to the simple mass-action prediction.

#### (ii) With undirected CTL motion, when CTL are sufficiently motile that handling time becomes limiting, clustered infections are more difficult to control than diffuse infections

For more rapid surveillance, in diffuse infections C* is given by mass-action and is independent of *I*_0_, while S : H ≫ 1. Mass-action eventually breaks down when CTL appear so late that infected cell densities are high and the search time approaches the handling time (figure 2*f–j*, blue curves). The search time 1/*kI*_0_ (*I*_0_ expressed as a proportion of all surveyable cells) is approximately 140 min at *I*_0_ = 1000, 70 min at *I*_0_ = 2000 and 35 min at *I*_c_ = 4000, compared to the handling time of 30 min. At this point, handling time begins to be limiting and C* starts to increase with the infected cell count (figure 2*h–j*, blue curves).

For very motile CTL and clustered infections, within foci the search time is very short (∼ 1/*k* ≃ 90 s) and is much less than the handling time. At low-infected cell numbers, at C* few CTL are required to kill more than once to bring the infection under control and the limiting effect of handling time is only weakly apparent (figure 2*f*). However, the low S : H ratio within clusters means that handling time becomes limiting at lower infected cell numbers than in the diffuse case (figure 2*g–j*, orange curves).

Thus, when CTL move with undirected random walks and are relatively efficient such that the time taken to move between adjacent cells is shorter than the handling time, the spatial distribution of infected cells can have a substantial impact on critical CTL densities. Clustered infections require more CTL to control than a diffuse infection growing at the same rate, and become progressively harder to control the later CTL arrive.

### (c) CTL chemotaxis reduces C* and the degree of chemotaxis influences the effect of spatial structure on the efficiency of clearance

**Figure 3.**
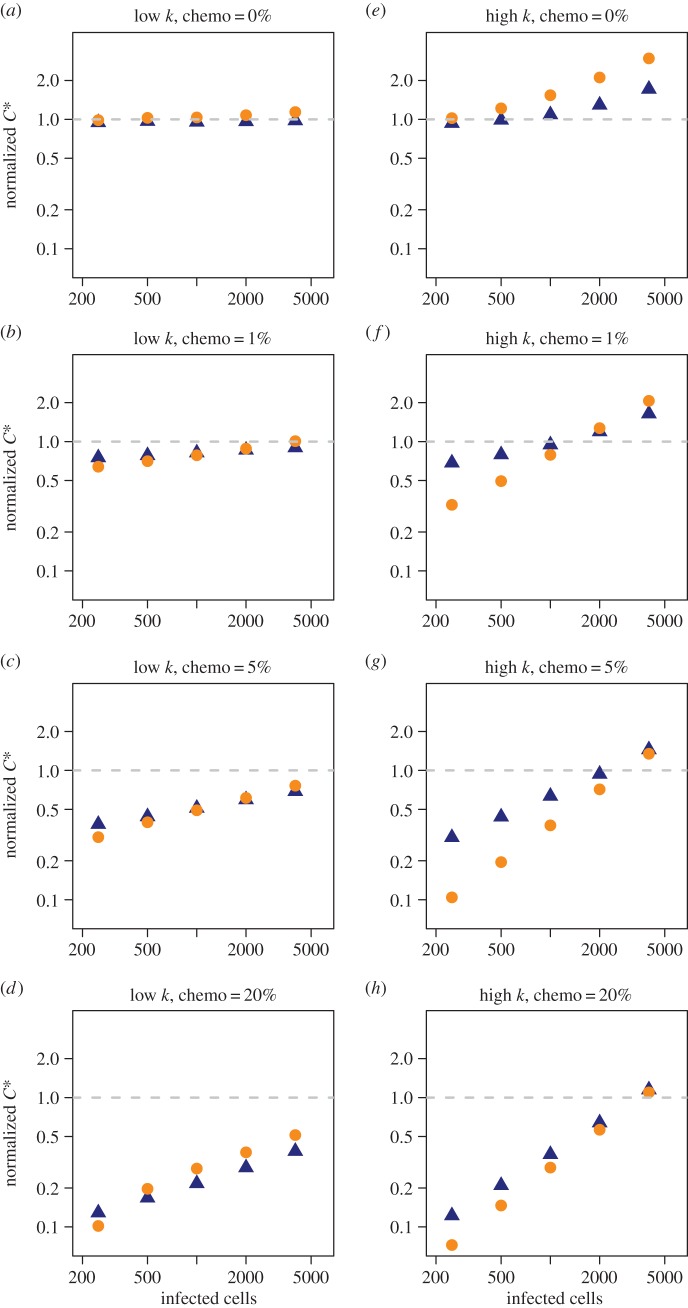
Critical CTL densities (in units of the basic mass-action estimate C*= *r*/*k*) for different levels of chemotaxis. (*a–d*) Slow CTL surveillance and (*e–h*) rapid surveillance. Blue triangles, diffuse infection; orange circles, clustered infection. Note logarithmic scale on *y*-axes.

Next we broke another implicit assumption of mass-action models and allowed different degrees of chemoattraction of CTL towards their targets. As described in Methods, this was done by assigning different probabilities that, at each turning event, a CTL moves in the direction of the nearest infected cell. This is a rather extreme implementation of chemotaxis that imposes no range on the influence of chemokine gradients, and so the probability of a turn being directed was limited to 20% at most. Unsurprisingly, chemotaxis reduces the number of CTL required for control in general (figure 3). This can be understood as an effective increase in the surveillance rate *k*. For all degrees of chemotactic attraction, the earlier CTL appear the more substantial the reduction in C* relative to undirected searches. This is because the sparser the targets are, the bigger is the fractional reduction in search time provided by directed motion towards infected cells.

At low surveillance rates (figure 3*a–d*) then, as with undirected motion, C* is insensitive to the spatial distribution of the infection for all levels of chemotaxis (blue and orange points lie close together). For both clustered and diffuse infections, S : H > 1 for inefficient CTL (the shortest search time between infected cells is 80 min even with highly directed motion, compared to a handling time of 30 min).

At higher surveillance rates (figure 3*e–h*), we see more complex behaviour that arises from the interplay between the positive effect of chemotaxis and the sequestration of CTL in conjugates that limits the killing rate. In general, we see that when CTL appear early, moderate levels of chemotaxis render clustered infections easier to control (figure 3*f–g*, left-hand points) but that differences in C* due to spatial structure shrink again as CTL motion becomes highly directed (figure 3*h*). Again, these results can be understood intuitively. Early in clustered infections, a directed search strategy substantially reduces the time for each CTL to locate a cluster. Subsequent location of infected cells is rapid and limited only by handling time. By contrast, early in diffuse infections the mean time for a randomly chosen CTL to locate its first target may be shorter than for a focal infection, but the identification of subsequent targets requires further migration. The net effect at moderate levels of chemotaxis is then that small focal infections are easier to control (figure 3*f–g*, left-hand points). However, at higher densities of infected cells (i.e. if CTL appear later) the time to locate the next target in either scenario becomes dominated by the handling time. Spatial structure then becomes less important (figure 3*f–g*, right-hand points). Finally, when migration is highly directed (figure 3*h*), the search time is small compared with handling time at all infected cell densities and for all spatial distributions, and so again the critical CTL densities for diffuse and clustered infections converge (figure 3*h*). As above, the fold reduction in C* is greatest the earlier CTL appear, because the fold increase in the search time is greatest when targets are sparse.

In summary, in this modelling framework (i) highly directed motion is an optimal search strategy for all patterns of infection; (ii) with any level of directed motion, the earlier CTL arrive at the site the fewer are required to clear the infection; (iii) in general, at weak to moderate levels of chemotaxis, the more clustered an infection the easier it is to control; and (iv) at very high levels of chemotaxis, such that locating targets is very efficient and handling time is limiting, the efficiency of clearance becomes insensitive to the spatial distribution of targets.

## 4. Discussion

The E : T ratio is typically considered the quantity of interest for the control of infections, but when considering the impact of spatial structure, we found that a key parameter was instead the ratio of the expected time to detect the next target (the search time) to the handling time, or S : H. Surprisingly, when CTL are inefficient in finding targets even within clusters (i.e. when S : H > 1) we found that the spatial distribution of targets has minimal impact on critical CTL densities. In this case, the simple mass-action prediction C* = *r*/*k* derived from microscopic parameters applies. When CTL are able to locate targets rapidly enough that handling time becomes limiting, we found (perhaps surprisingly) that the more clustered an infection the more CTL are needed for control. Chemotaxis towards infected cells decreased the critical density of CTL, most dramatically when CTL appear early in infections. Weak chemotaxis reversed the trend observed in undirected walks, and the more clustered an infection the fewer CTL are needed to control it. Finally, strongly directed motion towards infected cells renders the efficiency of control insensitive to the spatial structure of an infection.

Clearly, strongly directed motion is an optimal strategy for locating a given target rapidly. Neutrophils have been seen to flock rapidly towards foci of infection (see, e.g. [44] and references therein); Kastenmuller *et al.* [45] found strong evidence of chemokine-driven motion of CTL towards infection sites within the subcapsular sinus of a lymph node; and chemokines have been shown to drive the attraction of CTL, at least broadly, to tumour masses [46,47]. There is also evidence that the gp120 HIV envelope glycoprotein mediates chemoattraction of CTL via the CXCR4 chemokine receptor at low concentrations but there is repulsion at higher concentrations *in vitro*, potentially inhibiting CTL activity [48]. However to our knowledge, only two studies have directly quantified the statistics, and in particular the directedness, of effector CTL movement within an infected tissue *in vivo*. Harris *et al.* [49] described CTL motion as a Lévy flight, with persistent segments of lengths drawn from a heavy-tailed distribution, linked by turns through uniformly distributed angles. They observed an increase in CTL speed in the presence of infection but with random walk statistics unchanged (a phenomenon known as orthotaxis). However, the spatial distribution of targets was not characterized, and if CTL and targets are well mixed it may be difficult to detect a signature of directed motion on small scales using multiphoton imaging, particularly if it is weak [50–53]. Kelemen *et al.* [54] compared the trajectories of antigen-specific and non-specific T cells in the livers of mice infected with sporozoites of the malaria parasite and showed a propensity for cells of both types to move towards infected cells at distances up to 140 μm, or roughly six to seven hepatocyte widths. Interestingly, they saw a small but significant increase in the degree of attraction of antigen-specific over non-specific cells over distances of 40–140 μm but no difference in degrees of attraction at shorter distances. Again, this may be due to difficulties in detecting directed motion over such short length scales.

Much of our understanding of T-cell movement patterns comes from studies of naive T cells in lymph nodes, in the presence and absence of cognate antigen on dendritic cells (DC). Chemokines have been shown to attract naive CD8^+^ T cells towards DC [55–58], but other analyses have found little evidence for directed motion in the presence of antigen [39,59,60] and found that on small scales T cells perform persistent random walks, i.e. linear motion on short scales punctuated by turns through uniformly distributed angles. Again, though, directed motion may be undetectable if antigen-presenting cells are present at a high density and diffusely spread within a lymph node. It has been suggested that an undirected random walk is an optimal search strategy for naive T cells in the presence of cognate antigen [39,61], and a cellular automata model has been used to argue that directed motion may increase the occlusion of DC by non-specific T cells and hinder the efficiency of recruitment of antigen-specific cells into a response [62]. More recently however, a model of migration using cells with deformable membranes (the Cellular Potts Model) was used to argue the converse, showing that weak chemotaxis may indeed facilitate repertoire scanning [53].

Our analyses are most relevant for infections of relatively static cell populations in planar environments, such as layers of epithelial cells, interacting with an initially scattered population of CTL such as tissue-resident memory cells. Significant target motility will generate more diffuse patterns of pathogen spread and so will make the simple mass-action estimate more appropriate. We speculate that in three dimensions the search time is more likely to be limiting and so the simple mass-action estimate of C* may again be more appropriate, at least for the case of unbiased random walks. A plausibility argument is that the probability of locating a given target a finite distance away from a CTL is roughly equivalent to the drunkard's walk problem, in which one estimates the probability that a random walk will eventually return to its starting point. For random walks with constant step length, the probability of eventual return to the vicinity of the origin is 1 in two dimensions, but only approximately 0.34 in three dimensions [63].

In summary, our study shows that the average search time and the handling time are the key indicators of whether spatial structure needs to be considered when making estimates of critical CTL densities. In particular, we found that in the absence of chemotaxis and when handling time is not limiting, the mass-action estimate of C* applies, irrespective of spatial structure. This also illustrates that the inverse problem of inferring spatial structure from the kinetics of clearance is not well defined—the accuracy of the mass-action estimate of C* does not imply spatial homogeneity, and conversely, spatial heterogeneity does not imply that the mass-action estimate of C* is incorrect. Further, our simulations confirm the intuitive result that chemotactic motion of initially scattered CTL towards their targets appears to be an optimal strategy. What is striking is that, at least in planar environments, even weak chemotaxis can improve the efficiency of control markedly, and most notably for infections that are clustered. For example, with plausible parameters for CTL motility and the infection growth rate, a 1% probability that the next turn is directed towards an infected cell has the potential to reduce critical CTL numbers roughly threefold over the simple estimates early in infection (figure 3*f*). Since it seems clustering of infected cell populations is the norm, and that it is likely that CTL experience at least some degree of chemoattraction towards foci of infection, then our simulations suggest that a simple mass-action model derived from two microscopic parameters (uncontrolled pathogen growth rate and CTL surveillance rate) yields an upper bound on critical CTL densities; combined with orthotaxis and non-lytic modes of pathogen suppression, infections may be controlled with lower numbers of tissue-resident CTL than predicted with simple models.

## Supplementary Material

Supplementary Table 1
