# Supplementary material for: How is the effectiveness of immune surveillance impacted by the spatial distribution of spreading infections?
Source: Philos Trans R Soc Lond B Biol Sci. 2015 Aug 19;370(1675):20140289. doi: 10.1098/rstb.2014.0289 (PMC4528487; doi:10.1098/rstb.2014.0289)
Supplement: Supplementary Table 1 [file rstb20140289supp1.pdf]

Supplementary material for Kadolsky & Yates, 2015. The effect of spatial distribution of target cells on the efficiency of immune control. *Phil. Trans. R. Soc. B.* doi: 10.1098/rstb.2014.0289

Agent	Parameter name	Description	Default Value	Units
CTL	r_ctlspeed	Speed of CTL (straight line speed). For the High E:T runs where the surveillance rate (k) was slow, CTL speed was reduced by a factor of ~40.	0.125 (efficient)<br>0.003 (inefficient)	μm s^{-1}
	r_ctlpersist	Persistence length of CTL straight line movement (note: interrupted whenever the CTL scans a cell)	25	μm
	t_ctlscan	Time spent scanning cells to determine if infected	5.0	s
	t_ctlhandle	Time spent handling an infected cell before it is lysed	30	min
	CHEMOTAXIS_PC	Percentage chance of CTL heading towards nearest infected cell when choosing a new direction	0.0	-
Virion	r_vinfect [S]	Chance (per second) that an infected cell will infected a susceptible cell anywhere on the grid	1.74e-05	s^{-1}
	r_vspeed [C]	Virion straight line speed	1	μm s^{-1}
	r_vinfect [C]	Infection chance of the virion on encounter with susceptible cell (100%)	1	-
	r_death	Viron clearance rate (half life of ~4 hours)	4.63e-05	s^{-1}
Infected cells	r_ideath	Infected cell death rate, mostly due to viral cytotoxicity (half life of ~1.4 days).	5.79e-06	s^{-1}
	r_vproduction [C]	Production rate of 'virions' from an infected cell (~1.5 virions/day)	1.75e-05	virions s^{-1}
(Non-)Susceptible cells	nc_spacing	Spacing between (non-)susceptible cells when initialising the simulator	0	μm
	nc_susceptiblepc	Percentage of cells which are susceptible when initialising the simulator	25	(%)
	nc_totalcount	Total number of (non-)susceptible cells to place during simulator initialisation	100000	-

Supplementary Table 1: List of parameters and values for runs of the pathogen-host simulator. Legend. S: for the sparse model of infection only; C: for the cluster model of infection only.

© The Authors under the terms of the Creative Commons Attribution License http://creativecommons.org/licenses/by/3.0/, which permits unrestricted use, provided the original author and source are credited.
